# Genetic bases of C7 deficiency: systematic review and report of a novel deletion determining functional hemizygosity

**DOI:** 10.3389/fimmu.2023.1192690

**Published:** 2023-05-25

**Authors:** Andrea Balduit, Anna Monica Bianco, Alessandro Mangogna, Anna Maria Zicari, Lucia Leonardi, Bianca Laura Cinicola, Martina Capponi, Alberto Tommasini, Chiara Agostinis, Adamo Pio d’Adamo, Roberta Bulla

**Affiliations:** ^1^ Institute for Maternal and Child Health - IRCCS “Burlo Garofolo”, Trieste, Italy; ^2^ Department of Maternal and Child Health and Urological Sciences, Sapienza University of Rome, Rome, Italy; ^3^ Department of Molecular Medicine, Sapienza University of Rome, Rome, Italy; ^4^ Department of Medical, Surgical and Health Science, University of Trieste, Trieste, Italy; ^5^ Department of Life Sciences, University of Trieste, Trieste, Italy

**Keywords:** complement component 7 (C7), complement system, primary immunodeficiency, complement deficiency, functional hemizygosity

## Abstract

Primary complement system (C) deficiencies are rare but notably associated with an increased risk of infections, autoimmunity, or immune disorders. Patients with terminal pathway C-deficiency have a 1,000- to 10,000-fold-higher risk of *Neisseria meningitidis* infections and should be therefore promptly identified to minimize the likelihood of further infections and to favor vaccination. In this paper, we performed a systematic review about clinical and genetic patterns of C7 deficiency starting from the case of a ten-year old boy infected by *Neisseria meningitidis B* and with clinical presentation suggestive of reduced C activity. Functional assay *via* Wieslab ELISA Kit confirmed a reduction in total C activity of the classical (0.6% activity), lectin (0.2% activity) and alternative (0.1% activity) pathways. Western blot analysis revealed the absence of C7 in patient serum. Sanger sequencing of genomic DNA extracted from peripheral blood of the patient allowed the identification of two pathogenetic variants in the C7 gene: the already well-characterized missense mutation G379R and a novel heterozygous deletion of three nucleotides located at the 3’UTR (c.*99_*101delTCT). This mutation resulted in an instability of the mRNA; thus, only the allele containing the missense mutation was expressed, making the proband a functional hemizygote for the expression of the mutated C7 allele.

## 1 Introduction

Primary complement (C) deficiencies represent approximately 1-10% of all reported primary immunodeficiencies (PIDs) (1), even though markedly higher numbers were documented in specific national registries (2). The most prevalent C deficiencies affect mannose-binding lectin (MBL), which is estimated to occur in more than 10% of the Caucasian population, and C2 (3). Nevertheless, the vast majority of C deficiencies has an estimated prevalence of about 0.03% in the general population (4, 5). However, due to the redundancies in the immune system and to the frequent presence of asymptomatic patients, many C disorders remain undiagnosed.

C defects are usually inherited in an autosomal recessive pattern (except for C1-inhibitor deficiency, which is autosomal dominant, and properdin deficiency, which is X-linked) (6, 7) and can be mainly distinguished in: early C deficiencies regarding the classical, lectin or alternative pathway, defects in the terminal C components, and deficiencies affecting C regulatory components and receptors (8). Symptoms can broadly vary from almost none to serious and even lethal infections (9). Apart from inherited origin, C deficiencies may also be acquired through C overconsumption by immune complexes, reduced hepatic synthesis, increased protein loss *via* urine, presence of autoantibodies, and somatic mutations (1, 10).

The manifestations of inherited C deficiencies fall broadly into three different clinical scenarios, depending on which factor is missing: i) increased susceptibility to recurrent bacterial infections, due to inadequate opsonization, defective cell lysis, and/or onset of immune complex diseases, is usually associated to abnormalities in alternative, lectin and terminal pathways (11); ii) concomitant features of autoimmunity, especially systemic lupus erythematosus, can be observed in classical pathway defects (12); iii) dramatically enhanced or uncontrolled C activation leading to other immune-related disorders is mainly due to deficiencies in C regulators (13). As a general trend, these deficiencies result into severe sinopulmonary bacterial infections, bacteremia, and/or meningitis, mainly due to encapsulated bacteria (*i.e., Streptococcus pneumoniae*, *Haemophilus influenzae* type b, *Neisseria meningitidis*). In particular, recurrent neisserial infections can indicate possible terminal component deficiencies (C5-C9) (8) and early alternative pathway deficiency (properdin) (14).

Even in the presence of clinical manifestations, the diagnosis of C deficiencies is quite challenging due to their rarity, the heterogeneity in clinical symptoms and the complexity of diagnostic procedures. Conventionally, the activity of each activation arm of the C is initially tested by functional assays based on hemolytic activity (CH50, LP50, AH50) (3). Therefore, methods based on enzyme-linked immunosorbent assay (ELISA) have been developed to test the functionality of all three C pathways (15), followed by the quantification of specific individual components.

Due to the close association between genetic variants and C defects, genetic approaches are increasingly becoming part of the diagnostic path to unveil C deficiencies (4). In particular, gene sequencing has become a robust and common tool for confirming single component deficiencies detected through quantitative and functional assays, but also an alternative and immediate diagnostic procedure when functional tests are not easily available. Moreover, functional and protein analysis are time-consuming, less accurate and dependent on C system sensitivity to freeze-thaw cycles (6). Despite the analysis may be complicated by copy number variations, point mutations and pseudogenes, the introduction of next generation sequencing and the improvements in genetic tools are gradually changing the approach to the diagnosis of inborn errors of immunity.

In the current paper, we aimed at exploiting the promising potentialities offered by genetic workup as a support to the currently available immunological assays used to identify C deficiencies. We analyzed the case of a ten-year-old boy presenting C7 deficiency, reporting a novel mutation responsible for functional hemizygosity, and then performed a systematic review of the literature to unveil the genetic bases of C7 deficiency.

## 2 Patient and methods

### 2.1 Patient presentation

A ten-year-old boy was admitted to the pediatric emergency room of the Policlinico “Umberto I” (Rome, Italy), with fever (38.8°C), vomiting, headache, lethargic status and petechial rashes on the trunk and the right knee.

Lumbar puncture revealed clear, colorless cerebrospinal fluid (CSF), detecting normal glucose and protein levels. CSF was negative to the FILMARRAY™ meningitis/encephalitis test, a panel for the simultaneous identification of the fourteen pathogens most commonly associated to meningitis or encephalitis. However, positivity for *Neisseria meningitidis* (serogroup B) was detected in his blood. Past medical history and familiar anamnesis were unremarkable. Four years earlier, the patient received the tetravalent polysaccharide meningococcal vaccine and two doses of the 4-component meningococcal serogroup B vaccine. The patient recovered without sequelae after being treated with dexamethasone, ceftriaxone and inotropic agents for 14 days. High-flow oxygen therapy was also required.

### 2.2 Sample collection

Blood samples from the patient, his parents and brother were collected at the Policlinico “Umberto I” (Rome, Italy) for further analysis. Written informed consent for genetic analysis of all samples was obtained from parents.

### 2.3 Complement pathway functionality

The functionality of classical, alternative and lectin pathways was assessed by the commercial kit Wiesslab® (Technogenetics, Milan, Italy) (15), following the manufacturer’s instructions. The absorbance was read at 405 nm using the PowerWave X Microplate Reader (Bio-Tek Instruments). The absorbance of the Blank (Diluent) was subtracted from the absorbances of the negative control (NC, a serum lacking C activity), positive control (PC, a serum with normal C activity) and the samples. The PC absorbance should be >1.0 and the NC absorbance <0.2, after subtraction of the Blank. After calculating the mean OD405nm values for the sample, PC and NC, the percentage of C activity was calculated as follows:


$$\%\text{ C activity}=\frac{\left(\text{Sample}-\text{NC}\right)}{\left(\text{PC}-\text{NC}\right)}\times 100$$

### 2.4 Western blot analysis of complement components

Patient serum and a pool of normal human sera (NHS) were diluted (1:50) in Laemmli buffer. After boiling, 20 µL of samples were loaded on a 10% polyacrylamide gel and separated by SDS-PAGE under reducing conditions. For the specific analysis of C7 expression, 15, 10 and 5 µL of samples were also loaded and compared with 20 µL of C7 deficient human serum (Sigma). Proteins were transferred to a nitrocellulose membrane using the semi-dry Trans-blot Turbo Transfer System (BIORAD). A 1h-blocking step with 5% skimmed milk in Tris-Buffered Saline + Tween 20 (TBST, 10 mM Tris, pH 8.0, 150 mM NaCl, 0.5% Tween 20) was followed by an ON incubation at 4°C with primary antibodies, anti-C5 biotinylated (1:2,000, A306, Quidel), anti-C6 (1:1,000, A307, Quidel), anti-C7 (1:2,500, A308, Quidel), anti-C8 biotinylated (1:1,000, A708, Quidel) or anti-C9 (1:1,000, A310, Quidel). The following day, the membrane was incubated with anti-goat or streptavidin LI-COR IRDye 800CW (1:10,000; LI-COR Biosciences, Lincoln, NE, USA), for 1h at RT. The fluorescence intensity was acquired by the Odyssey® CLx near-infrared scanner (LI‐COR Biosciences, Lincoln, NE, USA). Image acquisition, processing and data analysis were performed with Image Studio Ver 5.2 (LI-COR Biosciences).

### 2.5 DNA extraction, PCR and prediction of functional effects of genomic variants

Genomic DNA was obtained from peripheral blood using the QIAsymphony workstation and the QIAsymphony DNA kits (Qiagen). DNA quality and quantity were assessed with agarose gel and Nanodrop 2000 spectrophotometer (ThermoFisher Scientific, Walrham, MA, USA). After genomic DNA isolation, the entire coding and flanking regions of C7 gene were amplified by KAPA2G FAST HS RM (Sigma-Aldrich) and PCR products were sequenced by Sanger sequencing. All sequences were analyzed with codon code aligner software (V.7.1.1 version). Only rare variants (minor allele frequency<1%) were considered as probably deleterious. To predict whether each variation could affect protein function, the following bioinformatics tools were used: Polyphen2 (http://genetics.bwh.harvard.edu/pph2/), Sift (http://sift.jcvi.org), Mutation Assessor (http://mutationassessor.org/r3/), Human Splicing Finder (http://www.umd.be/HSF/) and Transfact (http://gene-regulation.com/pub/databases.html#transfac). Controls were obtained from the Genome Aggregation Database (gnomAD), a reference population database of individuals with no manifested pathologies, stratified by regions of origin.

### 2.6 RNA extraction and RT-PCR

Total RNA was extracted from whole blood using the PAXgene Blood RNA kit (PreAnalytiX, Hombrechikon, Switzerland, produced by QIAGEN), according to the manufacturer’s instruction. The obtained RNA was quantified using Nanodrop 2000 spectrophotometer (ThermoFisher Scientific, Walrham, MA, USA). After isolation, total RNA was reverse-transcribed into cDNA using Transcriptor First Strand cDNA Synthesis Kit (Roche) and cDNA obtained was used as a template for Sanger Sequencing on C7 coding sequence obtained after PCR amplification by KAPA2G FAST HS RM (Sigma-Aldrich).

### 2.7 Study search strategy and selection

**Figure 1:**
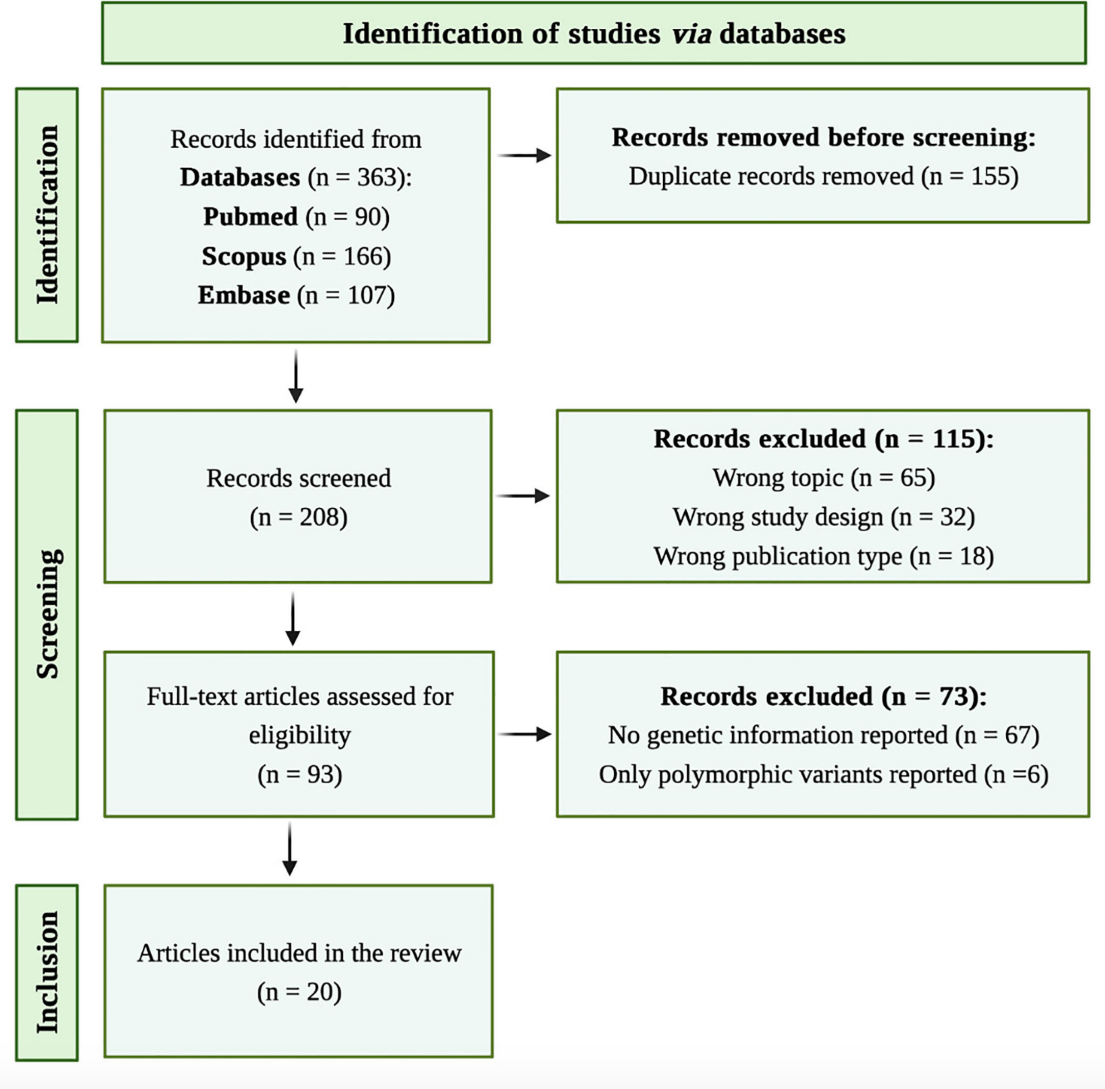
Flow diagram for study selection, according to PRISMA statement.

A systematic literature review was conducted to examine the already published information about mutations associated to C7 deficiency. The search strategy and article selection process are reported in the flowchart illustrated in **Figure 1**, according to the recommendations of the PRISMA guidelines (16). We performed initial literature search by scanning PubMed, Scopus and Embase databases for relevant articles published until 26^th^ January 2023. The search terms included C7 and deficiency in title, abstract and keywords. The specific search strategy is presented in the **Supplementary Material**. Only peer-reviewed articles accepted for publication and written in English language were included.

Reference lists of included studies were manually screened for eligible articles to ensure a comprehensive search. Screening of records and removal of duplicates were performed using Rayyan systematic review tool (17). Two independent investigators (AB and AM) screened and included the relevant articles if they met all of the following eligibility criteria: 1) case reports/series/original articles including the terms for C7 and deficiency; 2) English language; and 3) provision of sufficient information about the identification of novel mutations associated to C7 deficiency. Articles were not included if they were as follows: 1) reviews, meta-analysis, conference or comment papers; and 2) *in vitro* or animal studies. Disparities were resolved with a third investigator (AMB).

## 3 Results

### 3.1 Immunological characterization of the patient

The patient was referred to the Pediatric Immunology Unit of the Policlinico “Umberto I” (Rome, Italy), where a complete workup for suspected immunodeficiency was performed, including blood counts, immunoglobulin levels and vaccine titers. As shown in **Table 1**, complete immunological testing displayed normal values, except for lymphocyte subset which showed a low CD4+/CD8+ ratio. Since the clinical manifestations determined a high index of suspicion of C deficiency, C components’ levels and pathway activity were evaluated by the standard diagnostic protocol comprising the measurement of C3 and C4 and the analysis of total C activity.

**Table 1:** Immunophenotyping of circulating leukocytes of the patient by flow cytometry analysis.

Immunophenotype	Patient value	Reference range
% CD3+ CD45+ T Lymphocytes	78%	55 - 84%
CD3+ T Lymphocytes	2502 cells/μL	690 - 2540 cells/μL
% CD3+ CD4+ CD45+ T Lymphocytes	**20.83%**	31 - 60%
CD3+ CD4+ T Lymphocytes	631 cells/μL	410 - 1590 cells/μL
% CD3+ CD8+ CD45+ T Lymphocytes	**57.33%**	13 - 41%
CD3+ CD8+ T Lymphocytes	**1737 cells/μL**	190 - 1140 cells/μL
% CD3-/CD16+ CD56+ CD45+ NK Cells	9.07%	5 - 27%
CD3-/CD16+ CD56+ NK Cells	304 cells/μL	90 - 590 cells/μL
% CD19+ B Lymphocytes	10.47%	6 - 25%
B Lymphocytes	352 cells/μL	90 - 660 cells/μL
CD4+/CD8+ ratio	**0.36**	0.6 - 2.8

Patient values falling outside reference range were reported in bold.

C3 and C4 fractions were found to be in the normal range during the meningococcal sepsis, being 91.4 mg/dl (normal range: 90-180 mg/dl) and 30 mg/dl (normal range: 10-40 mg/dl), respectively. The assessment of C activity by CH50 was performed almost one month following sepsis and highlighted a dramatically low activity (9%; normal range: 50-150%).

### 3.2 Evaluation of complement pathway functionality

The serum of the patient was then tested for the functionality of the three C pathways using the Wieslab® Complement System Kit, an ELISA-based assay which enables the determination of the specific activity of all three pathways independently. This immunoassay has been validated for the semi-quantitative evaluation of C pathways’ functionality and the determination of C deficiencies in human serum. The assay detects C activation combining the principles of the hemolytic assay with the use of labelled antibodies specific for neoantigens. The amount of neoantigen produced is proportional to the functionality of C pathways (15).

During the incubation of the patient serum in the wells, C is activated by the specific coating. After washing, the amount of C5b-9 neoantigen detected with a specific alkaline phosphatase-labelled antibody is used as a readout of MAC (Membrane Attack Complex) formation.

The normal distribution within 2 standard deviations (SD) has been determined to be 69-129% of the PC for the qualitative assay. Values below the 69-129% range are indicative of either increased C activation and consumption, or a genetically determined low activity. Values lower than 5% strongly suggest a complete C deficiency due to either excessive activation or inherited deficiency in the classical C pathway. Our results indicated that the functionality of all three C pathways was completely absent in the patient’s serum (**Table 2**).

**Table 2:** Percentage of functional complement system pathways evaluated by ELISA test in patient’s family.

	AP (%)	CP (%)	MP (%)
Patient	**0.1**	**0.6**	**0.2**
Mother	76.6	107.6	55.3
Father	68.9	112.9	109.2
Brother	79.9	93.2	77.5

**Reference values:** 100-40% normal/39-11% questionable/<10% deficit.

AP, alternative pathway; CP, classical pathway; MP, MBL lectin pathway.

Since the pattern of inheritance of C deficiencies is usually autosomal recessive, whereas heterozygous carriers remain clinically silent, C activity analysis were extended also to the other members of the family. We observed a normal functionality of all three C pathways in both parents and brother (**Table 2**).

### 3.3 Evaluation of terminal complement proteins

Since the results obtained by the routine screening assays for the evaluation of C pathway integrity and activation confirmed the suspicion for a PID of the C and the clinical manifestations suggested the specific deficiency of the terminal pathway, single C factors in the serum of the patient were analyzed in order to identify the defective component. Thus, we evaluated the presence of C5, C6, C7, C8 and C9 by Western Blot analysis.

**Figure 2:**
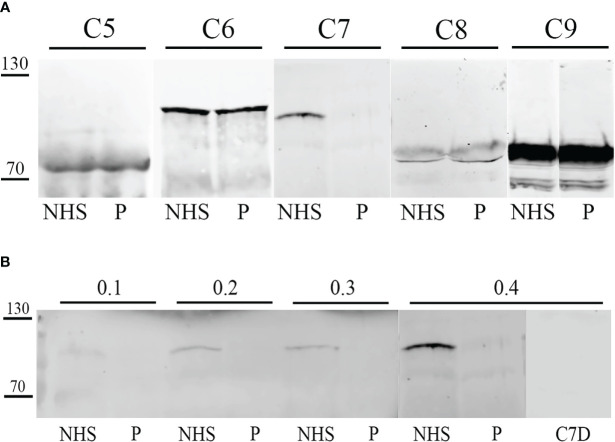
Western blot analysis of terminal complement **(C)** components in patient serum. Patient serum (P) and control serum (NHS) were tested by Western blot for the detection of terminal C components. After SDS-PAGE and transfer, membrane was probed with anti-C5, anti-C6, anti-C7, biotinylated anti-C8 and anti-C9 primary antibodies, followed by incubation with IRDye 800CW streptavidin or secondary antibody. Signal intensity was detected using an Odyssey CLx near-infrared scanner (LI-COR Biosciences, Lincoln, NE, USA). Image acquisition, processing and data analysis were performed with Image Studio 5.2 (LI-COR Biosciences). **(A)** Patient serum (P) and control serum (NHS) were compared for C5, C6, C7, C8 and C9 concentrations. **(B)** Patient serum (P) and control serum (NHS) were compared for C7 concentration by testing different serum dilutions (0.1-04 µL). Moreover, a C7-depleted (C7D) serum was used as a control.

Western Blot results showed the absence of C7 expression as compared to the control sample (NHS), whereas the other C proteins analyzed resulted as normally expressed (**Figure 2**).

### 3.4 Genetic analysis

**Figure 3:**
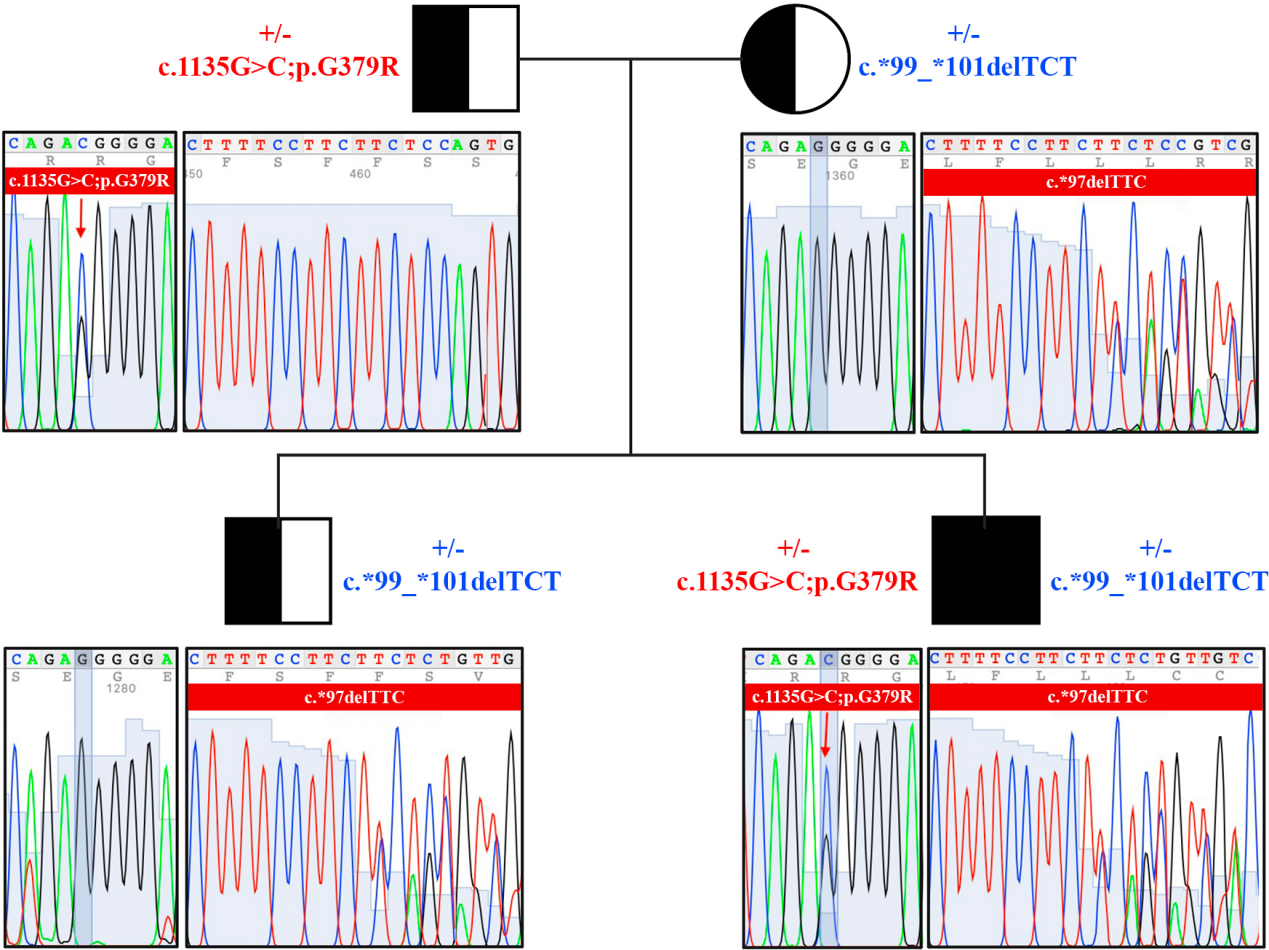
Pedigree and sequencing results of the family of C7-deficient patient. Genomic DNA was isolated from peripheral blood of the patient and of his relatives. Two pathogenetic variants were identified in the family members (c.1135G>C;p.G379R, and c.*99_*101delTCT).

Samples of genomic DNA extracted from peripheral blood of the patient and of his relatives were analyzed by Sanger sequencing performed on the coding region and intron/exon junctions of C7 gene. Nine variants were identified in the patient, including five at the intronic level (**Supplementary Table 1**). Among the intronic variants, two variants (rs1450656 in homozygosity and rs2876849 in heterozygosity) have already been reported as benign, while the other three were not reported in any public database, therefore no prediction was available. On the contrary, three variants in the coding region of the gene were already recorded in databases, two benign (rs1063499 in homozygosis and rs13157656 in heterozygosis) and one pathogenetic (rs121964921 in heterozygosis). The latter, also known as the pathogenetic variant G379R, was also looked for in genomic DNA samples taken from the patient’s parents and brother. The proband’s father was found to be a carrier of this variant, which, therefore, he passed on to his son (**Figure 3**).

Interestingly, a heterozygous deletion of three nucleotides in a region downstream of the stop codon was also detected in the proband, being named c.*99_*101delTCT. This deletion was located on the untranslated region 3’UTR of the cDNA. It has never been reported before and a possible deleterious effect is not known yet. To investigate the pattern of inheritance of this mutation, we analyzed its presence in the other family members, and we identified that the proband’s mother and brother were carriers of the deletion (**Figure 3**).

**Figure 4:**
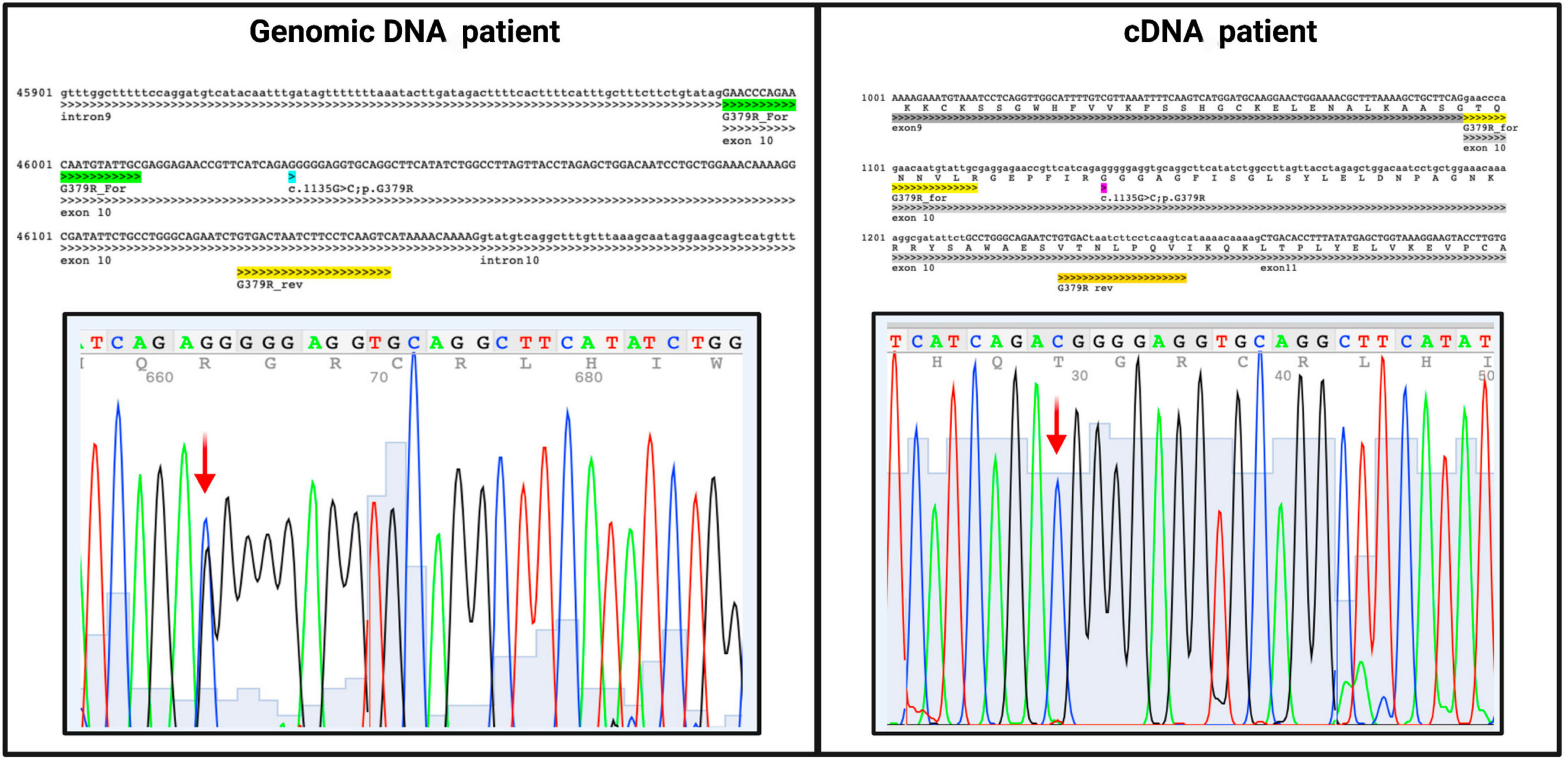
Sequencing results of both patient’s genomic and cDNA with the same pair of primers that align within exon10. The c.1135G>C variant is present in heterozygous in genomic sequence and in hemizygosity in cDNA, indicating only the father’s allele presence while the mother’s allele remained undetectable.

To evaluate whether this deletion could induce instability on C7 mRNA and determine its subsequent degradation, the patient’s mRNA was reverse-transcribed, and the cDNA was sequenced. Despite the very low expression of this gene in the whole blood cells, we were able to successfully amplify the coding region. The sequence analysis revealed the exclusive presence of the missense mutation within the region of the gene where the paternal mutation is located, while the other allele remained undetectable. This outcome is consistent with our hypothesis that the deletion caused mRNA instability, resulting in the expression of only the allele containing the missense mutation. Consequently, the proband emerged as a functional hemizygote for the expression of the mutated C7 allele within this specific gene region (**Figure 4**). The novel variant was registered in ClinVar database (Variation ID: SCV003920905).

### 3.5 Search results

The systematic search resulted into a total of 363 articles. After removal of duplicate reports *via* Rayyan, 208 articles were screened. Full-text screening resulted in 93 articles which were assessed for eligibility (**Figure 1**). Twenty studies were included in this systematic review. The identified mutations and, when available, patient clinical characteristics were described in **Table 3**.

**Table 3:** Characteristics of the mutations reported by the articles included in the systematic review.

Reference	Location of C7 defect	Defect	Zygosity	Type of mutation	Age (Sex)	Manifestation	Pathogen	History of infection/systemic diseases	Family history of infection	CH50
Shamriz et al., 2022 (18)	exon 10	c.1135G>C; p.G379R	hom	missense	14 (M)	MEN	*N. meningitidis*	no	n.a.	undetectable
	exon 10	c.1135G>C; p.G379R	hom	missense	9 (M)	MEN	*N. meningitidis*	yes (MEN)	yes	undetectable
Cummings et al., 2021 (19)	exon 12	c.1561C>A; p.R521S	n.a.	missense (associated to C6 variant, rs76202909)	0 (F)	central nervous system manifestations	neonatal herpes simplex virus	n.a.	n.a.	n.a.
Kageyama et al., 2021 (20)	intron 4	c.281-1G>T	het	splice acceptor variant	51 (M)	acute-onset high fever	*N. gonorrhoeae*	no	no	undetectable
	exon 11	c.1454C>T; p.A485V	het	missense						
El Sissy et al., 2019 (21)	exon 15	c.1924_1925delAG; p.H643Pfs*10	het	deletion	n.a.	n.a.	n.a.	n.a.	n.a.	n.a.
	exon 5	c.405delT; p.N136Tfs*2	hom/het	deletion	n.a.	n.a.	n.a.	n.a.	n.a.	n.a.
	exon 6	c.479delG; p.S160Ifs*20	het	deletion	n.a.	n.a.	n.a.	n.a.	n.a.	n.a.
	exon 7	c.633_643del; p.S212Hfs*4	het	deletion	n.a.	n.a.	n.a.	n.a.	n.a.	n.a.
	exon 11	c.1410delG; p.T471Pfs*22	het	deletion	n.a.	n.a.	n.a.	n.a.	n.a.	n.a.
	exon 13	c.1743delT; p.F581Lfs*15	het	deletion	n.a.	n.a.	n.a.	n.a.	n.a.	n.a.
	exon 4	c.189T>G; p.C63W	hom	missense	n.a.	n.a.	n.a.	n.a.	n.a.	n.a.
	exon 10	c.1135G>C; p.G379R	hom/het	missense	n.a.	n.a.	n.a.	n.a.	n.a.	n.a.
	exon 12	c.1561C>A; p.R521S	hom/het	missense	n.a.	n.a.	n.a.	n.a.	n.a.	n.a.
	exon 8	c.928G>A; p.G310R	het	missense	n.a.	n.a.	n.a.	n.a.	n.a.	n.a.
	exon 10	c.1117G>A; p.G373R	hom	missense	n.a.	n.a.	n.a.	n.a.	n.a.	n.a.
	exon 4	c.193G>T; p.G65*	hom	nonsense	n.a.	n.a.	n.a.	n.a.	n.a.	n.a.
	exon 16	c.2107C>T; p.Q703*	hom	nonsense	n.a.	n.a.	n.a.	n.a.	n.a.	n.a.
	intron 17	c.2350 + 1delG(IVS17 + 1)	het	splice variant	n.a.	n.a.	n.a.	n.a.	n.a.	n.a.
	intron 4	c.280 + 1G>A(IVS4 + 1)	het	splice variant	n.a.	n.a.	n.a.	n.a.	n.a.	n.a.
	intron 4	c.281-1G>T(IVS5-1)	hom	splice variant	n.a.	n.a.	n.a.	n.a.	n.a.	n.a.
	intron 17	c.2350 + 2T>C(IVS17 + 2)	hom/het	splice variant	n.a.	n.a.	n.a.	n.a.	n.a.	n.a.
Rosain et al., 2017 (22)	exon 4	c.189T>G; p.C63W	hom	missense	n.a.	n.a.	n.a.	n.a.	n.a.	n.a.
	exon 4	c.193G>T; p.G65*	hom	nonsense	n.a.	n.a.	n.a.	n.a.	n.a.	n.a.
	intron 4	c.280 + 1G>A	het	splice acceptor variant	n.a.	n.a.	n.a.	n.a.	n.a.	n.a.
	intron 4	c.281-1G>T	hom	splice acceptor variant	n.a.	n.a.	n.a.	n.a.	n.a.	n.a.
	exon 5	c.405delT; p.D136Tfs*2	hom	deletion	n.a.	n.a.	n.a.	n.a.	n.a.	n.a.
	exon 6	c.449delA; p.Q150Rfs*30	het	deletion	n.a.	n.a.	n.a.	n.a.	n.a.	n.a.
	exon 7	c.633_643del; p.S212Hfs*4	het	deletion	n.a.	n.a.	n.a.	n.a.	n.a.	n.a.
	exon 10	c.1135G>C; p.G379R	hom/het	missense	n.a.	n.a.	n.a.	n.a.	n.a.	n.a.
	exon 11	c.1410delG; p.T471Pfs*22	het	deletion	n.a.	n.a.	n.a.	n.a.	n.a.	n.a.
	exon 12	c.1561C>A; p.R521S	hom/het	missense	n.a.	n.a.	n.a.	n.a.	n.a.	n.a.
	exon 17	c.2350 + 2T>C; p.A784Lfs*25	het	insertion	n.a.	n.a.	n.a.	n.a.	n.a.	n.a.
	intron 17	c.2350 + 2T>C	het	splice variant	n.a.	n.a.	n.a.	n.a.	n.a.	n.a.
Sanges et al., 2017 (23)	exon 7	c.633_643del; p.S212Hfs*4	het	deletion	18 (M)	MEN	*N. meningitidis Y*	no	no	49%
	exon 12	c.1561C>A; p.R521S	het	missense						
Thomas et al., 2012 (24)	exon 8/9	c.739 + 1262_1270-2387delinsGCAGGCCA; p.S247fsX27	hom	deletion/insertion	n.a.	n.a.	n.a.	n.a.	n.a.	n.a.
Barroso et al., 2010 (25)	exon 15	c.2107C>T; p.Q681X	hom	nonsense	20 (M)	MEN	*N. meningitidis*	yes (MEN)	no	undetectable
	exon 2	c.90G>A; p.W8X	hom	nonsense	16 (F)	MEN	*N. meningitidis*	yes (MEN)	no	undetectable
	exon 9	c.1135G>C; p.G357R	het	missense						
Rameix-Welti et al., 2007 (26)	exon 11	c. 1561C>A; p.R499S	het	missense	18 (F)	MEN	*N. meningitidis*	n.a.	n.a.	8%
	exon 9	c.1135G>C; p.G357R	het	missense						
	exon 11	c. 1561C>A; p.R499S	het	missense	22 (F)	MEN	*N. meningitidis*	yes (MEN)	n.a.	23%
	intron 16	IVS16 + 2T>C	het	splice variant						
	exon 15	c.2107C>T; p.Q681X	hom	nonsense	N.A. (M)	MEN	*N. meningitidis*	n.a.	n.a.	<10%
	exon 9	c.1135G>C; p.G357R	het	missense	18 (F)	MEN	*N. meningitidis A*	yes (MEN)	n.a.	<10%
	exon 14	c.1924delAG; p.S620fs10	het	deletion						
	exon 3	c.189T>G; p.C41W	hom	missense	5 (M)	MEN	*N. meningitidis*	yes (MEN)	n.a.	<10%
	exon 9	c.1135G>C; p.G357R	hom	missense	17 (F)	MEN	*N. meningitidis*	yes (MEN)	n.a.	<10%
	exon 9	c.1135G>C; p.G357R	hom	missense	22 (F)	MEN	*N. meningitidis B*	yes (*H. parainfluenzae* MEN)	n.a.	<10%
	exon 12	c.1741–3delT; p.F569fs14	het	deletion	33 (F)	MEN	*N. meningitidis Y*	yes (MEN)	n.a.	<10%
	intron 16	IVS16 + 2T>C	het	splice variant						
	intron 16	IVS16 + 2T>C	hom	splice variant	(F)	MEN	*N. meningitidis*	n.a.	n.a.	<10%
Barroso et al., 2006 (27)	exon 10	c.1458T>A; p.C464X	n.a.	missense	32 (F)	malar erythema associated with sun exposure	n.a.	yes (SLE) C4B deficiency	n.a.	undetectable
	exon 11	c. 1561C>A; p.R499S		missense						
	exon 6	c.631delACGTCGACAGA; p.T189X193	n.a.	deletion	23 (F)	n.a.	n.a.	yes (MEN with septic shock)	n.a.	undetectable
	exon 14	c.1922delAG/1923delGA/1924delAG; p.S620X630	het	deletion						
Kang et al., 2006 (28)	exon 10	c.1424G>A; p.C475Y	hom	missense	38 (F)	MEN, ART, endopthalmitis	n.a.	n.a.	brother C7 deficient	< 5 U/mL
	intron 4	c.281-1G>T	hom	splice variant	14 (M)	MEN	n.a.	n.a.	n.a.	< 5 U/mL
Ki et al., 2005 (29)	intron 4	c.281-1G>T	hom	splice variant	11 (F)	MEN	n.a.	n.a.	n.a.	undetectable
	exon 1-17	c.1-?_2350+?del	n.a.	large deletion						
Barroso et al., 2004 (30)	exon 10	c.1309delA/1310delA/1311delA/1312delA/1313delA/1314delA; p.K416X419	n.a.	deletion	17 (F)	MEN	n.a.	yes (MEN)	n.a.	undetectable
	exon 9	c.1135G>C; p.G357R	n.a.	missense						
	exon 14	c.1922delAG/1923delGA/1924delAG; p.S620X630	hom	deletion	(F)	n.a.	n.a.	yes (MEN)	brother C7 deficient	undetectable
	exon 14	c.1922delAG/1923delGA/1924delAG; p.S620X630	hom	deletion	(M)	n.a.	n.a.	yes (MEN)	brother C7 deficient	undetectable
Vázquez-Bermúdez et al., 2003 (31)	exon 6	c.615G>A; p.W183X	het	missense	15 (M)	n.a.	n.a.	yes (MEN)	sister C7 deficient	undetectable
	exon 9	c.1135G>C; p.G357R	het	missense						
Behar et al., 2002 (32)	exon 9	c.1135G>C; p.G357R	n.a.	missense	17 (M)	MEN	n.a.	yes (MEN)	brother C7 deficient	n.a.
Horiuchi et al., 1999 (33)	exon 14	c.1957G>T; p.E631X	hom	nonsense	25 (F)	MEN	n.a.	yes (MEN)	n.a.	undetectable
O ‘Hara et al., 1998 (34)	exon 7/8	n.a.	n.a.	deletion of ~6-8 kbp	17 (M)	MEN	n.a.	yes (MEN)	brother and sister C7 deficient	n.a.
Fernie et al., 1997 (35)	intron 1	IVS1, G-A, -1	het	splice acceptor variant	n.a.	n.a.	n.a.	n.a.	n.a.	n.a.
	exon 9	c.1135G>C; p.G357R	hom	missense	n.a.	n.a.	n.a.	n.a.	n.a.	n.a.
	exon 7/8	n.a.	n.a.	deletion of ~ 7.4 kbp	n.a.	n.a.	n.a.	n.a.	n.a.	n.a.
Nishizaka et al., 1996 (36)	exon 16	c.2250T>A; p.C728X	hom	nonsense	33 (M)	MEN, septic shock, intravascular coagulation, adrenocortical insufficiency, cardiac failure	*N. meningitidis B*	n.a.	sister with SLE	undetectable
	exon 15	c.2137delTG/2138delGT/2139delTG; p.V692L	hom	deletion	33 (M)	n.a.	n.a.	no	n.a.	undetectable
Fernie et al., 1996 (37)	exon 11	c. 1561C>A; p.R499S	n.a.	missense	n.a.	n.a.	n.a.	n.a.	n.a.	n.a.

ART, arthritis; hom, homozygosity; het, heterozygosity; MEN, meningitis; n.a., not available; SLE, systemic lupus erythematosus.

p.G357R is another common annotation of p.G379R. The mutation was reported in exon 9 (25, 26, 30–32, 35) or exon 10 (18, 21, 22) in different articles.

## 4 Discussion

Terminal C deficiencies are rare but, when diagnosed, they are commonly associated with recurrent neisserial infections. C7 deficiency is known to be the second most common among late C deficiencies (0.0041%) (38). C7 complete deficiency predisposes patients to an increased risk of bacterial infections, especially meningitis caused by *Neisseria meningitidis*, with high vulnerability during childhood and adolescence due to the lack of a protective antibody immunity, but also in adult life. Individuals with homozygous C7 deficiency display normal C-mediated opsonization and chemotaxis; nevertheless, the complete absence of detectable C7 leads to the incapability to generate the MAC and to the lack of serum hemolytic and bactericidal activity. Moreover, Schifferli et al. reported that about 10% of C7 and other deficiencies of late-acting C components (except C9) were also combined to immune complex diseases (39).

Due to the rarity and clinical significance of C7 deficiency, the aim of the current study was to report the case of a ten-year-old child with C7 deficiency, who was found to bear a novel mutation in C7 gene, and to perform a systematic review about the genetic bases of C7 deficiency to compare the genetic features of our case with those previously described in the literature. Accordingly, a systematic literature review was performed following the PRISMA guidelines and screening full text articles published by indexed journals in PubMed, Embase, or Scopus database. Eligible studies encompassed those investigating C7 deficiency and reporting identified genetic mutations. In total, we identified 20 articles, including 16 case reports/series, 2 original articles and 2 multicenter retrospective studies.

The gene for C7 spans about 80kb, being encoded by 18 exons, and is located on chromosome 5p13, which also encodes C6 and C9 (40); in fact, C7 deficiency was also found associated to C6 variants (19, 41, 42). Since the first description of a C7 deficiency case by Boyer et al. in 1975 (43), a hereditary pattern of autosomal codominance have been reported. Over the next few years, reports were confined to the characterization of the clinical course of C7 deficient patients, due to the absence of suitable genetic tools for identifying the mutations responsible for the deficient phenotype. Later, researchers focused on marker haplotype studies of both C6 and C7 genes, hypothesizing that they may pertain to a single genetic unit since the primary RNA transcript contains information for both proteins (44). The first genetic variants associated to C7 deficiency were identified only in the mid-1990s (36, 37).

The genetic bases for C7 deficiency are heterogeneous, since different families have been showed to carry distinct molecular defects. Interestingly, some genetic variants appeared to be homogeneous and prevalent in isolated geographical areas. In our systematic search, we identified 41 distinct variants associated to C7 deficiency. Missense variants were the most commonly found, whereas splice variants, insertions, duplications and deletions were less frequently detected. In particular, according to our systematic review of the literature, G379R and R521S appeared to be the most frequently reported pathogenetic point mutations.

In our patient, nine variants were identified, including five at the intronic level. In particular, we identified two pathogenetic variants, G379R and c.*99_*101delTCT. The single nucleotide variant G379R (also known as G357R), identified in heterozygosis in the proband and the father, was already reported and characterized in previous studies, being defined as pathogenetic and commonly associated to C7 deficiency (18, 21, 22, 25, 26, 30–32, 35). This sequence change leads to the replacement of glycine, which is neutral and non-polar, with arginine, which is basic and polar, at codon 379 of the C7 protein, being expected to disrupt C7 protein function. In a large cohort retrospective study by El Sissy et al., the presence of this variant was detected in 22% of patients affected by C7 deficiency (21). Moreover, C7 deficiency caused by the pathogenic variant G379R was reported in 1.1% of the Moroccan Jews in Israeli population (45).

Interestingly, a heterozygous small deletion in the region downstream of the stop codon was detected in the proband, being named c.*99_*101delTCT. The deletion has not been previously reported in any scientific publication or in public databases. Deletions in C7 gene were previously reported (21, 24, 27, 29, 30, 34, 36), but this is the first time that a deletion in C7 gene was found on 3’UTR of the gene. The 3′UTR is located downstream of the coding sequence, and it is involved in the regulation of mRNA-based processes, impacting on mRNA stability, transcription and localization, but exerting effects also on regulators like microRNAs and RNA-binding proteins and on protein secondary structure (46). An imbalance in these regulatory mechanisms is known to alter molecular pathways and cellular functions, potentially leading to pathological processes.

In the hypothesis that this deletion could induce instability on the C7 mRNA and its subsequent degradation, the patient’s mRNA was reverse-transcribed, and the cDNA was sequenced. Despite the low expression level of this gene in the whole blood, we successfully amplified the coding region. The sequence showed the presence of only the missense mutation whilst the other allele resulted undetectable. This result confirmed the initial hypothesis that the deletion could determine an instability of the mRNA; thus, only the allele containing the missense mutation was expressed, making the proband a functional hemizygote for C7 expression.

A deeper understanding of the genetic bases of the individual C deficiencies and the implementation of genetic workup for the correct and prompt identification of subjects with C deficiencies may be essential in order to significantly lower the risk of severe respiratory infections, sepsis and meningitis. An extensive description and systematic annotation of newly identified C variants in variant databases may support the interpretation of their clinical significance to disease. Even at the first episode of infection, genetic tools are therefore essential not only for diagnostic purposes and for a proper treatment of the patients, but also to preventively identify other family members at risk and favor vaccination.

## 5 Strengths and limitations

To our knowledge, this is the first systematic review of the literature focusing on the genetic variants associated to a C component deficiency. The strengths of the current search include a comprehensive coverage of the available literature and ready-to-use information accessibility. However, we recognize some methodological limitations of the present systematic search. First, not all studies may be included in bibliographic databases, being unpublished conference papers or congress proceedings. Moreover, some genetic variants can be submitted to variant databases (*e.g.*, ClinVar), but not be described in published papers. Lastly, only studies published in English were selected, potentially introducing language bias.

## 6 Conclusion

We identified a novel variant of C7 gene (c.*99_*101delTCT), which determined mRNA instability and functional hemizygosity. Due to the close association between genetic variants and inherited C defects, genetic approaches are increasingly becoming part of the diagnostic path to unveil immunodeficiencies and to preventively identify family members at risk. The high recurrence of neisserial infections in patients affected by deficiencies in late components of the classical C pathway determines an urgent need for a deeper comprehension of the genetic bases of C deficiencies.

## Data availability statement

The original contributions presented in the study are included in the article/**Supplementary Material**. Further inquiries can be directed to the corresponding author.

## Author contributions

Conceptualization: CA, AA, and RB. Methodology: AB, AMB, AM, and CA. Resources: AZ, LL, BC, and MC. Data curation: AB, AMB, and AM. Writing—original draft preparation: AB, AMB, AM, and CA. Writing—review and editing: AZ, AT, AA, and RB. Supervision: AA and RB. Funding acquisition: AT, AA, and CA. All authors have read and agreed to the submitted version of the manuscript. All authors contributed to the article.
