# Supplementary material for: Genetic bases of C7 deficiency: systematic review and report of a novel deletion determining functional hemizygosity
Source: Front Immunol. 2023 May 25;14:1192690. doi: 10.3389/fimmu.2023.1192690 (PMC10248053; doi:10.3389/fimmu.2023.1192690)
Supplement: Supplementary file 1 [file DataSheet_1.docx]

*Supplementary Material to*

Genetic bases of C7 deficiency: systematic review and report of a novel deletion determining functional hemizygosity

**Andrea Balduit^1$^, Anna Monica Bianco^1$^, Alessandro Mangogna^1$^, Anna Maria Zicari^2^, Lucia Leonardi^2^, Bianca Laura Cinicola^2,3^, Martina Capponi^2^, Alberto Tommasini^1,4^, Chiara Agostinis^1^*^†^, Adamo Pio d’Adamo^1,4†^ and Roberta Bulla^5†^**

^1^ Institute for Maternal and Child Health - IRCCS “Burlo Garofolo”, Trieste, Italy

^2^ Department of Maternal and Child Health and Urological Sciences, Sapienza University of Rome, Rome, Italy

^3^ Department of Molecular Medicine, Sapienza University of Rome, Rome, Italy

^4^ Department of Medical, Surgical and Health Science, University of Trieste, Trieste, Italy

^5^ Department of Life Sciences, University of Trieste, Trieste, Italy

**^$^These authors contributed equally to this work and share first authorship**

**^†^These authors contributed equally to this work and share senior authorship**

*** Correspondence:**Chiara Agostinis (cagostinis@units.it). Institute for Maternal and Child Health - IRCCS “Burlo Garofolo”, Trieste, Italy. Phone: +39 040 5588652.

**^Search strategy^**

| **Databases** | PubMed/Medline, Scopus, Embase | |
| --- | --- | --- |
| **Total** | 363 | |
| **Database** | **n** | **Search** |
| **PubMed** | 90 | (((((((((((C7 deficient[Title/Abstract]) OR (C7 deficiency[Title/Abstract])) OR (C7 deficiencies[Title/Abstract])) OR (seventh complement deficient[Title/Abstract])) OR (seventh complement deficiency[Title/Abstract])) OR (seventh complement deficiencies[Title/Abstract])) OR (7th complement deficient[Title/Abstract]) OR (7th complement deficiency[Title/Abstract])) OR (7th complement deficiencies[Title/Abstract])) OR (complement 7 deficient[Title/Abstract])) OR (complement 7 deficiency[Title/Abstract])) OR (complement 7 deficiencies [Title/Abstract]) |
| **Scopus** | 166 | (TITLE-ABS-KEY(c7 AND deficient) OR TITLE-ABS-KEY(c7 AND deficiency) OR TITLE-ABS-KEY(c7 AND deficiencies) OR TITLE-ABS-KEY(seventh AND complement AND deficient) OR TITLE-ABS-KEY(seventh AND complement AND deficiency) OR TITLE-ABS-KEY(seventh AND complement AND deficiencies) OR TITLE-ABS-KEY(7th AND complement AND deficient) OR TITLE-ABS-KEY(7th AND complement AND deficiency) OR TITLE-ABS-KEY(7th AND complement AND deficiencies) OR TITLE-ABS-KEY(complement 7 deficient) OR TITLE-ABS-KEY(complement 7 deficiency) OR TITLE-ABS-KEY(complement 7 deficiencies)) AND ( LIMIT-TO ( LANGUAGE,"English" ) ) AND ( EXCLUDE ( DOCTYPE,"re" ) ) AND ( LIMIT-TO ( EXACTKEYWORD,"Complement Component C7" ) ) |
| **Embase** | **107** | ('c7 deficient':ti,ab,kw OR 'c7 deficiency':ti,ab,kw OR 'c7 deficiencies':ti,ab,kw OR 'seventh complement deficient':ti,ab,kw OR 'seventh complement deficiency':ti,ab,kw OR 'seventh complement deficiencies':ti,ab,kw OR '7th complement deficient':ti,ab,kw OR '7th complement deficiency':ti,ab,kw OR '7th complement deficiencies':ti,ab,kw OR 'complement 7 deficient':ti,ab,kw OR 'complement 7 deficiency':ti,ab,kw OR 'complement 7 deficiencies':ti,ab,kw) AND [english]/lim |

**Supplementary Table 1. Variants identified in the C7 gene (NM_000587.4) of the patient by Sanger sequencing.**

| Variant | Effect | Zigosity | rs | MAF | Interpretation |
| --- | --- | --- | --- | --- | --- |
| c.983-230T>G | n.a. | hom | n.a. | n.a. | n.a. |
| c.983-9C>T | n.a. | hom | rs1450656 | C=0.35640 | benign |
| c.1135G>C | **p.G379R** | **het** | **rs121964921** | **C=0.00013** | **pathogenetic** |
| c.1166G>C | p.S389T | hom | rs1063499 | G=0.4300 | benign |
| c.1661+444A>T | n.a. | het | n.a. | n.a. | n.a. |
| c.1759A>C | p.T587P | het | rs13157656 | C=0.240352 | benign |
| c.1883-134A>G | n.a. | het | n.a. | n.a. | n.a. |
| c.2166-37A>T | n.a. | het | rs2876849 | T=0.3106 | benign |
| c.*99_*101delTCT | **n.a.** | **het** | **n.a.** | **n.a.** | **n.a.** |

**Abbreviations:** hom: homozygosis; het: heterozygosis; n.a.: not available; MAF: minor allele frequency.
